# Comparison of laparoscopic and robotic liver surgery: a German real-world analysis

**DOI:** 10.1007/s00464-025-12470-1

**Published:** 2026-01-05

**Authors:** P. H. von Kroge, M. Fard-Aghaie, K. Afshar-Bakshloo, J. R. Izbicki, T. Hackert, J. Li, I. Schmelzer, F. Nickel, T. Ghadban, A. Heumann

**Affiliations:** Department of General, Visceral and Thoracic Surgery, University Medical Center of Hamburg-Eppendorf, Martinistraße 52, 20246 Hamburg, Germany

**Keywords:** Robotic surgery, Liver surgery, Laparoscopic surgery, Liver tumor, Minimally invasive liver surgery

## Abstract

**Background:**

Robotic operations are becoming increasingly important in minimally invasive liver surgery. Although safety and efficiency have already been demonstrated, real-world data regarding robotic experiences compared to laparoscopic procedures remain limited.

**Methods:**

We conducted a retrospective analysis of all patients who underwent laparoscopic or robotic resection for malignant or benign liver tumors between January 2020 and June 2024 at the University Medical Center Hamburg-Eppendorf.

**Results:**

A total of 308 patients met the inclusion criteria. Of these, 133 patients underwent robotic resection with the DaVinci®-Xi system, while 175 patients were operated laparoscopically. In the robotic cohort, major resections were performed more frequently (27 [20.3%] vs. 14 [8.0%]; *p* = 0.002). Moreover, the rate of patients with liver cirrhosis was higher (34.6% vs. 20%; *p* = 0.004), and more patients were classified as ASA 3–4 (61.7% vs. 48.6%; *p* = 0.022). Additionally, a greater number of patients with hepatocellular carcinoma and biliary neoplasia underwent robotic resection (54.0% vs. 34.2%; *p* = 0.004). Operation time was significantly shorter in the laparoscopic group (132 min vs. 187 min; *p* = 0.004). There were no statistically significant differences between groups for conversion rate, intraoperative blood loss, overall complication rate, and complications classified as $\ge$ 3A according to the Clavien–Dindo system.

**Conclusion:**

In a real-world setting, we retrospectively observed a selection tendency favoring the robotic approach for multimorbid patients, particularly those with liver cirrhosis and primary malignant liver tumors with higher complexity, highlighting the evolving role of robotic surgery in managing complex liver resections.

**Graphical abstract:**

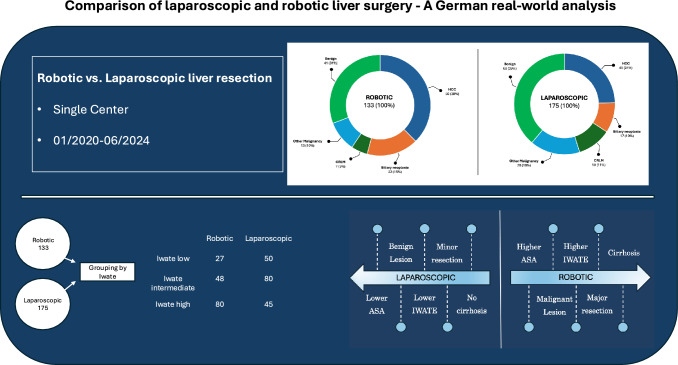

## Background

In recent years, various studies have demonstrated the advantages of minimally invasive liver surgery (MILS) [1]. The primary benefits include lower complication rates, reduced intraoperative blood loss, a shorter hospital stay, and comparable oncological outcomes [2–7]. As laparoscopic procedures have shown promising results, robotic approaches for all types of resection are becoming increasingly common. The safety, feasibility, and successful implementation of robotic programs at specialized centers have been frequently demonstrated. Nevertheless, the role of robotic access in MILS still requires a more precise definition [8, 9].

Recently, several studies have been published comparing laparoscopic and robotic approaches for MILS using propensity score-matched cohorts. Some of these studies demonstrated advantages of robotic approaches across various resection types [10–12], while others found no differences [13]. Furthermore, most studies have focused directly on comparing identical interventions, concerning their advantages and disadvantages, rather than defining the complementary roles of each method within MILS.

To address this gap, the present study aimed to shift the perspective from direct comparison of individual interventions to real-world data regarding the selection of patients for different surgical approaches. Therefore, we compared all patients undergoing MILS in our center in terms of intraoperative and postoperative short-term outcomes, as well as the selection criteria for the surgical approach.

## Methods

This single-center retrospective study included patients who met all the following criteria:

1. Age ≥ 18 years
2. MILS, either laparoscopic or robotic resection at University Medical Center Hamburg-Eppendorf, Germany, between January 2020 and June 2024. Patients who did not undergo formal liver resection were excluded from the study.

### Clinical data collection

MILS was introduced at our center in 2014 through the use of laparoscopy. Robotic liver resections were performed starting in 2018. As part of the program’s establishment, the respective specialized surgeons attended external training courses on MILS. Exclusion criteria for MILS were primarily perihilar cholangiocarcinomas and other hilar tumors with vascular involvement.

For the analysis, the patient collective was divided into two groups: Laparoscopic liver resection and robotic liver resection. The DaVinci® Xi surgical system (Intuitive Surgical, Sunnyvale, CA, USA) was used for the robotic approach.

All patients included in this study were treated at the University Medical Center Hamburg–Eppendorf, Germany, between January 2020 and June 2024.

Clinical treatment and disease characterization data were collected from the patient’s electronic medical records. Baseline demographic data, age, sex, body mass index (BMI), and physical status according to the American Society of Anesthesiologists (ASA) [14] were collected. Disease- and operation-specific data included blood loss, operation time, entity, extent of resection, complications, length of hospital stay, liver cirrhosis, and conversion rate. Complications were classified according to the Clavien–Dindo [15]. The Iwate criteria were used to compare the interventions in terms of degree of complexity [16]. The extent of liver resections was defined according to the Brisbane terminology [17]. Bile leakage was classified according to the ISGLS definition [18].

Data collection was performed in accordance with local legal requirements (§ Hamburgisches Krankenhausgesetz). The study was approved by the Medical Ethical Committee, approval number PV3548, Hamburg, Germany. All procedures performed in this study involving human participants were in accordance with the ethical standards of the institutional and national research committee and with the 1964 Helsinki Declaration and its later amendments or comparable ethical standards. The ethics committee waived informed consent since only anonymous data was analyzed and published.

### Surgical technique

Patients were routinely operated on in the reverse Trendelenburg position. In cases of laparoscopic resection of the right posterior segments, these patients were operated on in a modified lateral position using wedge pillows or a vacuum mattress. A capnoperitoneum with a maximum pressure of 14 mmHg was established. In cases of major resections or cases of higher complexity, a synthetic 5 mm band was placed around the hepatoduodenal ligament to enable a Pringle maneuver. If necessary, it was carried out for a duration of 15 min with 5 min breaks in between. Before resection, intraoperative ultrasound was performed to determine the resection margins and to assess the adjacent structures and vessels. In laparoscopic resections, parenchyma dissection was performed using a bipolar clamp or LigaSure™ (Medtronic, Dublin, Ireland). Laparoscopic stapler devices (Medtronic, Dublin, Ireland) or laparoscopic cavitron ultrasonic surgical aspirator (CUSA, Valleylab, CO, USA) were used in some exceptions. In robotic resections, transection was performed using a modified crush and clamp technique with bipolar clamps. Larger vessels were clipped or transected using vascular staplers in both approaches.

### Statistical analysis

All statistical analyses were performed using the Statistical Package for Social Sciences statistical software, version 29.0 (IBM Corp., Armonk, New York, USA). Continuous values are presented as median with interquartile range (IQR). Nominal variables are expressed as numbers (%) and compared by the Chi-square test. The correlation of categorical data was tested using the Chi-square test and the Kruskal–Wallis test when there were multiple categories. As appropriate, continuous data were tested using the Student’s t-test or the Mann–Whitney *U* test. A two-sided *p *value < 0.05 was considered statistically significant.

## Results

During the defined study period, a total of 51% (range 46–60%) of patients underwent MILS, which was primarily performed by four specialized surgeons. A total of 308 patients underwent laparoscopic or robotic liver resection. Of these, 175 were operated on laparoscopically, and 133 were treated robotically.

### Patients’ characteristics

In the laparoscopic cohort, 88 (50.3%) patients were female, and the median BMI was 26.6 (IQR 23.5–30.5) kg/m^2^. With 63 (47.4%) female patients and a median BMI of 26.2 (IQR 23.3–31.1) kg/m^2^ in the robotic group, there were no statistically significant differences between the two groups. The mean age was 62.5 (IQR 53.8–70) years and 61 (IQR 45–70) years, respectively.

The rate of patients with liver cirrhosis (Child–Pugh A and B) was significantly higher in the robotic cohort (34.6% vs. 20%; *p* = 0.004). Additionally, more patients were classified as ASA 3–4 (61.7% vs. 48.6%; *p* = 0.022). Regarding the operated entity, the two groups showed significantly different distributions. More patients with hepatocellular carcinoma and biliary neoplasia underwent robotic resection (54.0% vs. 34.2%). Regarding the benign lesions, the distribution was reversed, with 30.9% treated robotically and 38.9% operated laparoscopically.

**Fig. 1:**
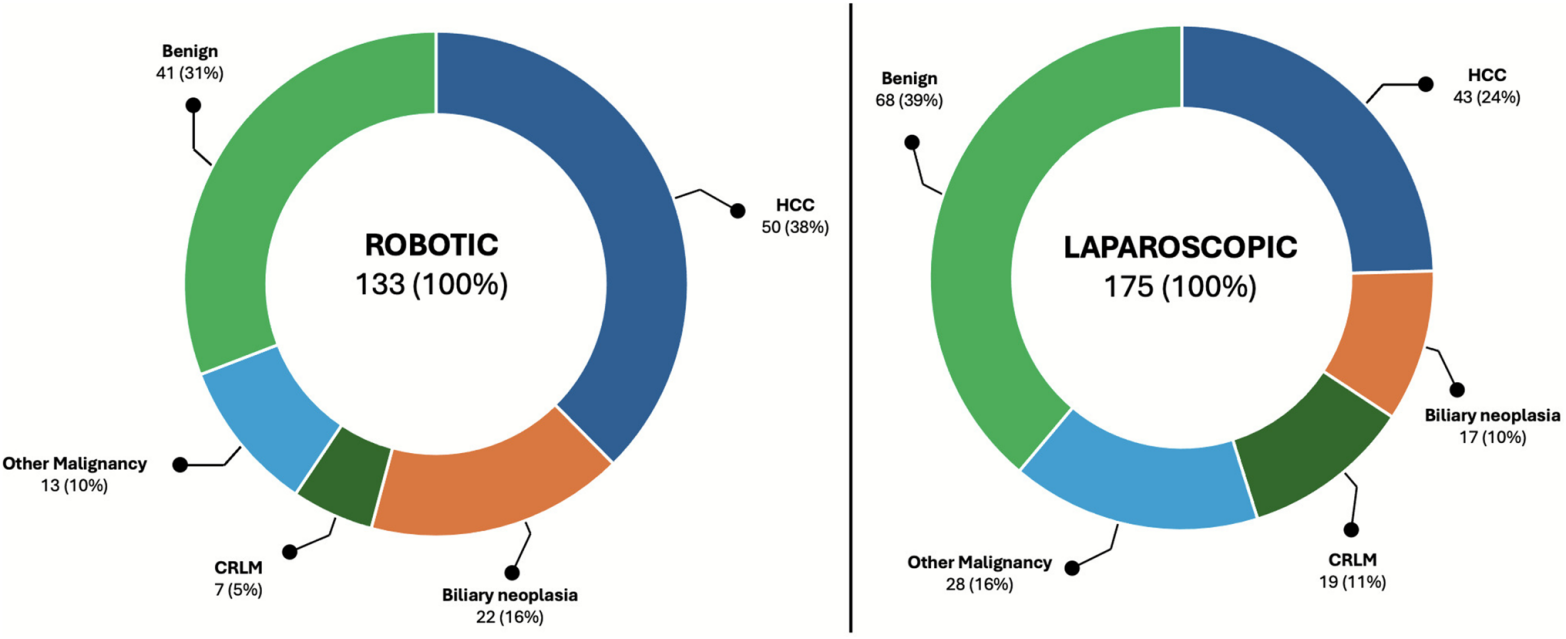
Distribution of the entities of both groups. The split figure with two pie charts illustrates the distribution of entities within the two groups, represented as *n* (%). The left diagram illustrates the distribution in the robotic group, while the right diagram shows the corresponding distribution in the laparoscopic group. The distribution between the groups differs significantly, with more primary hepatic malignancies in the robotic group and more benign indications and CRLM in the laparoscopic group (*p* < 0.005). *HCC* Hepatocellular carcinoma, *CRLM* Colorectal liver metastasis

Table 1 presents patients’ characteristics and demographic data. Figure 1 shows the distribution of the underlying tumor entities.

**Table 1:** Patients’ characteristics

	Robotic *(n* = *133)*	Laparoscopic (*n* = *175*)	*p* value
Age in years, median (IQR)	62.5 (53.8–70)	61 (45–70)	0.184
Sex, female (%)	63 (47.4)	88 (50.3)	0.612
ASA, *n* (%)			**0.022**
1–2	51 (38.3)	90 (51.4)	
3–4	82 (61.7)	85 (48.6)	
BMI (kg/m^2^), median (IQR)	26.2 (23.3–31.1)	26.6 (23.5–30.5)	0.431
Entity,* n* (%)			**0.004**
HCC	50 (37.6)	43 (24.5)	
Biliary neoplasia	22 (16.4)	17 (9.7)	
CRLM	7 (5.3)	19 (10.9)	
Other malignancy	13 (9.8)	28 (16.0)	
Benign lesion	41 (30.9)	68 (38.9)	
Cirrhosis			**0.004**
No cirrhosis, *n* (%)	87 (65.4)	140 (80.0)	
Child–Pugh A, *n* (%)	44 (33.1)	31 (17.7)	
Child–Pugh B, *n* (%)	2 (1.5)	4 (2.3)	
In hospital stay, median (IQR)	5 (4–7)	5 (3–6)	0.329
Mortality*,* *n *(%)	0 (0)	0 (0)	1.000

Statistically significant values (*p* < 0.05) are given in bold

*IQR* Interquartile range

### Operation details

All included patients underwent MILS. In the robotic group, 64 (48.1%) were atypical resections, and 69 (51.9%) were anatomic resections. With 90 (51.4%) atypical and 85 (48.6%) anatomic laparoscopic resections, there was no difference between the groups. The R0-resection rate was 88% in the robotic group and 92.6% in the laparoscopic group (*p* = 0.171). In the robotic cohort, major resections were performed more frequently (27 [20.3%] vs. 14 [8.0%]; *p* = 0.002). In addition, the Iwate score was higher in the robotic group (6 vs. 5; *p* = 0.002). With an operating time of 132 min (IQR 92–176), the operating time was shorter in the laparoscopic group. It was 187 min (IQR 142–261) in the robotic group (*p* = 0.004).

**Fig. 2:**
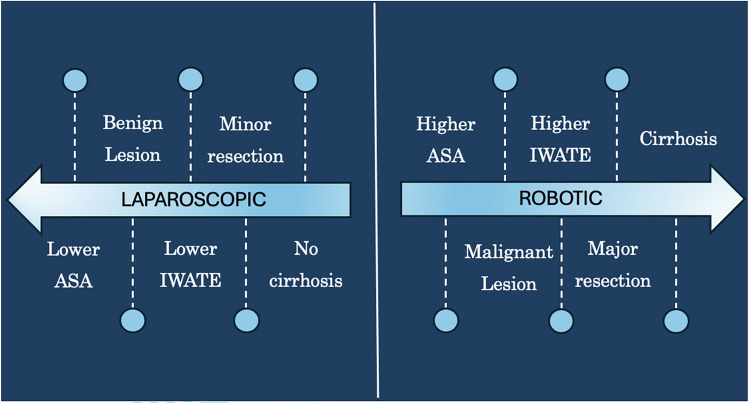
Factors influencing the operation approach. This split diagram, featuring two diverging arrows (left: laparoscopic, right: robotic) provides an overview of relevant factors that have shown an influence or a statistically significant difference between the two groups in our cohort. In cases with higher ASA, higher Iwate score, presence of cirrhosis, malignant lesion, and major resections, the robotic approach is more likely to be chosen. Laparoscopy, on the other hand, is more to be selected for minor resections and benign lesions in patients without cirrhosis, those with a lower ASA score, and those with an overall lower Iwate score

In total, bile leakage occurred in 7 patients. Of these, four patients had grade A, two had grade B, and one had grade C bile leakage according to ISGLS. There were no statistically significant differences between conversion rate, intraoperative blood loss, overall complication rate, and severity of complications. The length of in-hospital stays did not differ, with 5 (IQR 4–7) days after robot-assisted surgery and 5 (IQR 3–6**)** days after laparoscopic surgery (*p* = 0.329). Table 2 presents the operation details. Figure 2 illustrates the key factors influencing the choice of modality, as identified from our data.

**Table 2:** Operation details

	Robotic (*n* = *133*)	Laparoscopic (*n* = *175*)	*p* value
Resection type			0.170
Atypic, *n* (%)	64 (48.1)	98 (56.0)	
Anatomic, *n* (%)	69 (51.9)	77 (44.0)	
Extent of resection			**0.002**
Major, *n* (%)	26 (20.3)	14 (8.0)	
Minor, *n* (%)	107 (79.7)	161 (92.0)	
Conversion*, n (%)*	14 (10.5)	15 (8.6)	0.561
Major, n (%)	5 (19.2)	2 (14.2)	
Minor, n (%)	9 (8.4)	13 (8.1)	
Operation time in min, median (IQR)	187 (142–261)	132 (92–176)	**0.004**
Blood loss in ml, median (IQR)	500 (200–1100)	250 (100–1000)	0.065
Complications, *n (%)*	31 (23.3)	41 (23.4)	0.980
Clavien–Dindo classification			0.596
$<$ 3A	24 (18.0)	29 (16.6)	
$\ge$ 3A	7 (5.3)	12 (6.8)	
Bile leakage, *n* (%)	5 (3.8)	2 (1.1)	0.246
A	3	1	
B	1	1	
C	1	0	
R0-resection,* n* (%)	116 (88.0%)	162 (92.6)	0.171
Iwate-Score, median (IQR)	6 (4–8)	5 (3–7)	**0.002**
Iwate-subgroups,* n* (%)			**0.002**
Iwate low (0–3)	27 (20.3)	50 (28.6)	
Iwate intermediate (4–6)	46 (34.6)	80 (45.7)	
Iwate high (7–12)	60 (45.1)	45 (25.7)	

Statistically significant values (*p* < 0.05) are given in bold

*IQR* Interquartile range

For a detailed subgroup analysis and to reduce selection bias, the cohort was stratified according to the Iwate score, analogous to a recently published study on the benchmark outcome of robotic liver resection [19]. For this purpose, the cohort was divided into three groups: Iwate low (0–3), Iwate intermediate (4–6), and Iwate high (7–12). In the laparoscopic group, there were 50 (28.6%) patients in the Iwate low group, 80 (45.7%) in the Iwate intermediate group, and 45 (25.7%) in the Iwate high group.

In the robotic group, there were 27 (20.3%), 46 (34.6%), and 60 (45.1%) patients in the respective subgroups, with significantly more patients in the Iwate high group (*p* = 0.002).

In the Iwate low subgroup, there were more tumors > 3 cm (44% vs. 18%, *p* = 0.013), and a longer operation time (148 min [IQR 97–182] vs. 90 min [IQR 59–114], *p* = 0.001) in the robotic cohort compared to the laparoscopic cohort.

In the Iwate intermediate subgroup, there were more patients with cirrhosis (50% vs. 20%, *p* = 0.001), ASA 3–4 (63 vs. 38.7%, *p* = 0.009), and longer operation time (162 min [IQR 124–196] vs. 134 min [IQR 101–176], *p* = 0.003) in the robotic group. The CCI was higher in the laparoscopic cohort (*p* = 0.026), and the distribution regarding the tumor location differed significantly, with predominantly located tumors in segments II, VI, VII, and VIII in the laparoscopic group compared to segments IVa, V, VII, and VIII in the robotic group (*p* = 0.004).

In the Iwate high subgroup, the primary location of the tumors was predominantly in segments IVa, V, VII, and VIII in the robotic group. In contrast, tumors were located mainly in segments VII and VIII in the laparoscopic group (*p* = 0.001). In addition, tumors were > 3 cm more frequently (93.3% vs. 77.8%, *p* = 0.02) and more often close to major vessels (46.7% vs. 11.1%, *p* = 0.001) in the robotic group. As in the other subgroups, the operation time was longer in robotic surgery (250 min [IQR 186–276] vs. 171 min [IQR 140–223], *p* = 0.001). The patient outcomes and operation details stratified by Iwate score are presented in Table 3***.***

**Table 3:** Patient outcomes stratified according to Iwate score

	Iwate 0–3			Iwate 4–6			Iwate 7–12		
Group, *n*	Rob. n (%) 27 (20.3)	Lap. n (%) 50 (28.6)	*p*	Rob. n (%) 46 (34.6)	Lap. n (%) 80 (45.7)	*p*	Rob. n (%) 60 (45.1)	Lap. n (%) 45 (25.7)	*p*
Tumor location°, *n* (%)			0.345			**0.004**			**0.001**
III	8 (29.6)	13 (26.0)		1 (2.2)	1 (1.3)		–	–	
II, VI	8 (29.6)	23 (56.0)		8 (17.4)	27 (33.8)		2 (3.3)	–	
IVb, V	11 (40.8)	14 (28.0)		25 (54.3)	17 (21.4)		30 (50.0)	7 (15.6)	
I, IVa	–	–		2 (4.4)	8 (10.0)		7 (11.7)	7 (15.6)	
VII, VIII	–	–		10 (21,7)	27 (33.8)		21 (35.0)	31 (68.9)	
Cirrhosis, *n* (%)	7 (25.9)	11 (22.0)	0.698	23 (50)	16 (20.0)	**0.001**	16 (26.7)	8 (17.8)	0.283
ASA,* n (%)*			0.799			**0.009**			0.302
1–2	10 (37.0)	20 (40.0)		17 (37.0)	49 (61.3)		22 (36.7)	21 (46.7)	
3–4	17 (63.0)	30 (60.0)		29 (63.0)	31 (38.7)		38 (63.3)	24 (53.3)	
Resection type, *n (%)*			1.000			0.869			0.302
Minor	27 (100)	50 (100)		44 (95.6)	77 (96.2)		36 (60.0)	34 (57.6)	
Major	–	–		2 (4.4)	3 (3.8)		24 (40.0)	11 (42.4)	
Tumor > 3 cm,* n* (%)	12 (44.4)	9 (18.0)	**0.013**	17 (37.0)	44 (55.0)	0.051	56 (93.3)	35 (77.8)	**0.020**
Proximity to major vessel, *n* (%)	0 (0)	0 (0)	1.000	0 (0)	6 (7.5)	0.057	28 (46.7)	5 (11.1)	**0.001**
Hospital stay in days, median (IQR)	4 (4–5.5)	3 (2–6)	0.162	5 (4–6)	4 (3–6)	0.633	6 (5–8)	6 (4–8)	0.485
Blood loss in ml, median (IQR)	300 (100–550)	100 (50–275)	0.339	400 (200–600)	200 (100–900)	0.355	750 (375–1725)	750 (300–2000)	0.332
Liver failure ISGLS grade B/C, (%)	0 (0)	0 (0)	1.000	0 (0)	0 (0–0)	1.000	0 (0)	0 (0–0)	1.000
Conversion, *n* (%)	1 (3.7)	2 (4)	0.949	1(2.2)	8 (10)	0.101	10 (16.6)	5 (11.1)	0.421
Operation time in min, median (IQR)	148 (97–182)	90 (59–114)	**0.001**	162 (124–196)	134 (101–176)	**0.003**	250 (186–276)	171 (140–223)	**0.001**
Complications $\ge$ 3A*, *n* (%)	0 (0)	0 (0)	1.000	1 (2.2)	7 (8.8)	0.145	6 (10.0)	5 (11.1)	0.854
CCI, median (IQR)	0 (0–0)	0 (0–0)	0.235	0 (0–0)	0 (0–8.7)	**0.026**	0 (0–20.9)	0 (0–8.7)	0.653
R0, *n* (%)	26 (96.3)	48 (90)	0.949	42 (91.3)	74 (92.5)	0.811	49 (81.7)	40 (88.9)	0.308
Bile leakage, *n* (%)			0.171			0.693			0.314
A	1 (3.7)	0 (0)		0 (0)	1 (1.3)		2 (3.3)	0 (0)	
B	0 (0)	0 (0)		1 (2.2)	1 (1.3)		0 (0)	0 (0)	
C	0 (0)	0 (0)		-	0 (0)		1 (1.7)	0 (0)	
Mortality, *n* (%)	0 (0%)	0 (0%)	1.000	0 (0%)	0 (0%)	1.000	0 (0%)	0 (0%)	1.000

Statistically significant values (*p* < 0.05) are given in bold

^*^According to Clavien–Dindo classification

°according to Couinaud segments; *IQR* Interquartile range, *CCI* Comprehensive complication index; *Rob.* Robotic, *Lap.* Laparoscopic

## Discussion

In addition to classical open liver surgery and the already established laparoscopic surgical procedures, robot-assisted interventions have also recently gained importance. However, the exact role of laparoscopic and robotic surgery remains to be defined. By analyzing our single-center cohort of 308 patients, we identified differences in patient selection regarding comorbidities, resection type, and operation indication without finding differences in postoperative complications or length of in-hospital stay.

In our single-center cohort, the robotic group included significantly more patients with cirrhosis, higher ASA PS, and more patients who suffered from HCC or biliary tumors. Moreover, significantly more patients underwent major resections, and the procedures were considered to be of higher complexity overall, as indicated by the higher Iwate score. However, complication rates showed no statistical differences, indicating the safety and feasibility of robotic liver surgery. Notably, the bile leakage rate was similar, which aligns with the results of several other groups [20, 21].

A further subgroup analysis was performed using the Iwate score, with a grouping that was recently used to determine the benchmark cutoffs for robotic liver resections [19]. As indicated by the overall higher Iwate score, there were more Iwate high cases in the robotic group.

Despite longer operation time and more cases with larger tumors, there were no differences in patients with low Iwate (0–3). The longer operation time might partially be explained by larger tumors themselves and the necessary docking in robotic surgery. In contrast to our results, other propensity score-matched studies have also found advantages using robotic resection for minor resections or for resections in the anterolateral segments [12, 21]. This may be because, due to the Iwate criteria, our Iwate low subgroup only includes minor resections of the anterolateral segments without technically complex procedures, which are included in the other stratifications of the subgroups.

Advantages of robotic surgery were particularly demonstrated in the subgroup with intermediate and high Iwate scores. Despite having more patients with cirrhosis and a higher ASA, an overall lower CCI was shown, with a more complex distribution of segments in the intermediate group. Major complications and bile leakage were similar. Among the most complex cases, there were higher proportions of cases with larger tumors and vascular involvement. Here, too, no statistical differences were found regarding postoperative outcomes, particularly blood loss, R1 resection rate, and complication rate.

In comparison to the established benchmark cutoffs, our results of robotic resections meet the criteria regarding hospital stay, operation time, Liver failure, mortality, and CCI. For biliary leakage, the incidence is slightly increased in the intermediate group. This is mainly due to the cohort size, as there is only one case of leakage. Regarding major complications (Clavien–Dindo grade $\ge$ 3A), the rate is slightly higher in the Iwate high group. The R1 rates were slightly higher in the Iwate intermediate and Iwate high groups. The deviations from the benchmark cutoffs are clearly multifactorial. In contrast to the benchmark cohort, > 50% of cases in our cohort are primary malignant liver tumors, and no liver donor resections are included. Furthermore, with > 60% ASA 3–4, the cohort is overall sicker than the benchmark cohort [19].

Our results regarding the frequencies of the entities are partially in line with those of Sijberden et al. This is particularly evident in the more frequent HCC resections and fewer colorectal metastases observed in the robotic group [22]. In our cohort, primary hepatic tumors were significantly more often in the robotic group, while CRLM and benign lesions were more likely to be laparoscopically operated. Similar distributions could be shown in an additional Dutch multicenter national analysis [21].

In the robotic group, the mean operation time was prolonged.

Consistent with our results, longer operation times in robotic liver resections have been frequently reported in unmatched analyses [23, 24]. Comparing benchmark studies regarding laparoscopic liver resection and robotic liver resections, the operation time is described as shorter in laparoscopy, especially for cases with low and intermediate complexity. In fact, the median operation times are shorter than the cutoffs in all subgroups in our cohort [19, 25]. However, a recently published propensity score matched study showed no differences after stratifying according to the location of resection [22].

Overall, the patients operated robotically had a higher ASA PS, indicating higher morbidity of the collective. Other analyses yielded similar results in an unmatched cohort prior to propensity-score matching [21]. Furthermore, cirrhosis has already been identified as a factor for complexity in MILS [26]. Moreover, cirrhosis is associated with a longer postoperative course, higher postoperative morbidity, and increased blood loss [27]. As our results show, higher rates of liver cirrhosis have also been demonstrated in another multicenter retrospective analysis comparing robotic and laparoscopic liver resections [22]. More patients with cirrhosis might be due to the higher rate of HCCs as the selection criteria for the robotic approach.

As the subgroup analysis of our cohort confirmed, the degree of difficulty regarding tumor size and its anatomical location according to the Couinaud segments and proximity to major vessels is significantly higher in the Iwate intermediate and high group. In their single-center analysis of 600 patients, Schmelzle et al. also reported a higher Iwate score in their robotic group. Major resections, larger tumor size, and difficulty regarding tumor location differed significantly in comparison to the laparoscopic surgery collective [10]. Pilz da Cunha et al. reported similar trends with higher complexity, more major resections, and a higher rate of patients with cirrhosis without calculating the Iwate score in their cohort [21]. However, for larger tumors measuring more than 10 cm, a multicenter propensity score-matched analysis could not show advantages for either approach [13]. Despite the generally higher complexity of the procedures, the complication rates were similar in our cohort. Overall, our data confirm the current literature regarding patient selection for laparoscopic and robotic procedures in a real-world setting.

This study has some limitations that need to be discussed. The primarily retrospective design without randomization leads to a selection bias. However, it is important to note that one of the objectives of this study was to illustrate patient selection based on real-world data. Due to the primary stratification based on the surgical approach and not on a specific procedure with a possibly matched cohort, the distribution of procedures and entities is inconsistent between the groups. However, since the perspective relates to the indication and not to a direct comparison of the procedures in terms of their advantages and disadvantages, this cannot be avoided.

In conclusion, our real-world data point out that the robotic approach is more likely to be chosen for overall sicker patients with higher ASA PS and cirrhosis in more complex procedures and anatomical locations with a higher degree of difficulty. Advantages of robotic liver surgery have been demonstrated primarily for cases with intermediate or high Iwate score. With the same complication and conversion rate, the importance of robotics appears to be high in those cases, while technically simple procedures continue to be performed laparoscopically.
